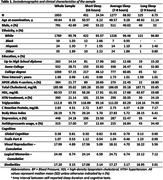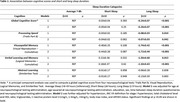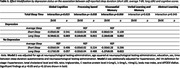# Sleep Duration and Cognitive Performance in Middle‐Aged and Older Adults: What is the Role of Depression?

**DOI:** 10.1002/alz.089859

**Published:** 2025-01-03

**Authors:** Vanessa M. Young, Rebecca Bernal, Andree‐Ann Baril, Joy Zeynoun, Crystal Wiedner, Alexa S Beiser, Matthew P. Pase, Jayandra J. Himali, Sudha Seshadri

**Affiliations:** ^1^ Glenn Biggs Institute for Alzheimer’s & Neurodegenerative Diseases, University of Texas Health Science Center, San Antonio, TX USA; ^2^ Graduate School of Biomedical Sciences, San Antonio, TX USA; ^3^ School of Social and Behavioral Sciences, Arizona State University, Phoenix, AZ USA; ^4^ Glenn Biggs Institute for Alzheimer’s & Neurodegenerative Diseases, University of Texas Health San Antonio, San Antonio, TX USA; ^5^ University of Montreal, Montreal, QC Canada; ^6^ Research Center of the CIUSSS‐NIM, Hôpital du Sacré‐Coeur de Montréal, Montreal, QC Canada; ^7^ The Framingham Heart Study, Framingham, MA USA; ^8^ Boston University School of Public Health, Boston, MA USA; ^9^ Boston University Chobanian & Avedisian School of Medicine, Boston, MA USA; ^10^ Harvard T.H. Chan School of Public Health, Harvard University, Boston, MA USA; ^11^ Turner Institute for Brain and Mental Health & School of Psychological Sciences, Monash University, Clayton, VIC Australia; ^12^ Department of Population Health Sciences, University of Texas Health Sciences Center, San Antonio, TX USA; ^13^ Boston University School of Medicine, Boston, MA USA; ^14^ Framingham Heart Study, Framingham, MA USA; ^15^ Department of Neurology, University of Texas Health Sciences Center, San Antonio, TX USA

## Abstract

**Background:**

Recent research has highlighted the importance of sleep on cognitive processes. However, conflicting evidence exists regarding optimal sleep duration and the impact of other co‐occurring conditions, such as depression. A diagnosis of depression in mid‐life may increase the risk of developing dementia. We examined the association between self‐reported sleep duration and cognition and whether depression status modified this relationship.

**Method:**

Dementia‐and‐stroke‐free participants 45 years and older from the Framingham Heart Study Third‐Generation, Omni 2, and New Off‐spring Cohorts were included (n = 1,853; age 49.8[SD 9.2] years; 42.69% male; **Table 1**). Neuropsychological testing assessed verbal learning and memory abilities, abstract reasoning skills, processing speed and visuospatial memory. Depression was defined as having CES‐D ≥16 or being under pharmacological treatment (n = 448; 32%). Multivariable linear regression models examined the association between sleep duration categories (≤6h; >6‐<9h [reference]; ≥9h), individual cognitive tasks and global cognition, adjusting for age, sex, education and time between sleep and cognitive assessments. A second model included further adjustment for vascular risk factors and *APOE4* status.

**Result:**

Long sleep duration (≥9h) was associated with worse global cognition (β±SE: ‐0.24±0.07; *p*<0.001) compared to average sleep duration. In cognitive domain‐specific tasks, long sleep was associated with worse verbal learning and memory abilities (‐1.50±0.60, *p* = 0.013), visuospatial memory (‐1.74±0.42, *p*<0.001), and processing speed (‐0.08±0.03, *p* = 0.014), but not with abstract reasoning skills (‐0.06±0.28, *p* = 0.838). Depression status significantly modified the association (global cognition int. p = 0.015; visual int. p = 0.006; and processing speed int. p = 0.038), where long sleep duration was associated with global cognition (‐0.34±0.11; *p* = 0.003), visuospatial memory (‐2.16±0.68; *p* = 0.002), and processing speed (‐0.14±0.05; *p* = 0.011) in those with depression. Long sleep duration was also associated with visuospatial memory in those without depression (‐1.27±0.55, p = 0.022) (**Table 3**). Short sleep duration (≤6h) was not associated with cognition (**Table 2**) and did not interact with depression status (**Table 3**).

**Conclusion:**

Long sleep duration was associated with worse cognition particularly among adults with depression, underscoring the complex sleep‐mood‐cognition interplay. Further research should explore the longitudinal impacts and causal mechanisms of suboptimal sleep. These findings may inform public health promotion of optimal sleep to maintain cognitive health among persons with depression.